# Publisher Correction: A conserved glutathione binding site in poliovirus is a target for antivirals and vaccine stabilisation

**DOI:** 10.1038/s42003-022-04378-6

**Published:** 2022-12-23

**Authors:** Mohammad W. Bahar, Veronica Nasta, Helen Fox, Lee Sherry, Keith Grehan, Claudine Porta, Andrew J. Macadam, Nicola J. Stonehouse, David J. Rowlands, Elizabeth E. Fry, David I. Stuart

**Affiliations:** 1 Division of Structural Biology, University of Oxford, The Henry Wellcome Building for Genomic Medicine, Headington, Oxford, OX3 7BN UK; 2 Magnetic Resonance Center CERM, University of Florence, Via Luigi Sacconi 6, 50019 Sesto Fiorentino Florence, Italy; 3 Department of Chemistry, University of Florence, Via della Lastruccia 3, 50019 Sesto Fiorentino Florence, Italy; 4 The National Institute for Biological Standards and Control, Potters Bar, EN6 3QG UK; 5 Astbury Centre for Structural Molecular Biology, School of Molecular and Cellular Biology, Faculty of Biological Sciences, University of Leeds, Leeds, LS2 9JT UK; 6 The Pirbright Institute, Pirbright, Surrey, GU24 0NF UK; 7 Diamond Light Source, Harwell Science and Innovation Campus, Didcot, OX11 0DE UK

**Keywords:** Structural biology, Cryoelectron microscopy

Correction to: *Communications Biology* 10.1038/s42003-022-04252-5, published online 25 November 2022.

In this article the citation number 62 referring to the open-source, non-commercial software PyMOL was incorrectly given as “Lilkova, E. et al. The PyMOL molecular graphics system, version 2.0 (Schrodinger, LLC., 2015)”, but should have been “The PyMOL Molecular Graphics System version 2.0 (Schrödinger, LLC, 2015).”.

These have now been corrected in the PDF and HTML versions of the Article.

