# Supplementary material for: A conserved glutathione binding site in poliovirus is a target for antivirals and vaccine stabilisation
Source: Commun Biol. 2022 Nov 25;5:1293. doi: 10.1038/s42003-022-04252-5 (PMC9700776; doi:10.1038/s42003-022-04252-5)
Supplement: Supplementary file 2 — Supplementary information [file 42003_2022_4252_MOESM2_ESM.pdf]

Supplementary Information

A conserved glutathione binding site in poliovirus is a target for antivirals and vaccine stabilisation

Mohammad W. Bahar*, Veronica Nasta, Helen Fox, Lee Sherry, Keith Grehan, Claudine Porta, Andrew J. Macadam, Nicola J. Stonehouse, David J. Rowlands, Elizabeth E. Fry, David I. Stuart*

*Correspondence and request for materials should be addressed to M.W.B (mohammad.bahar@strubi.ox.ac.uk) and D.I.S (dave.stuart@strubi.ox.ac.uk).

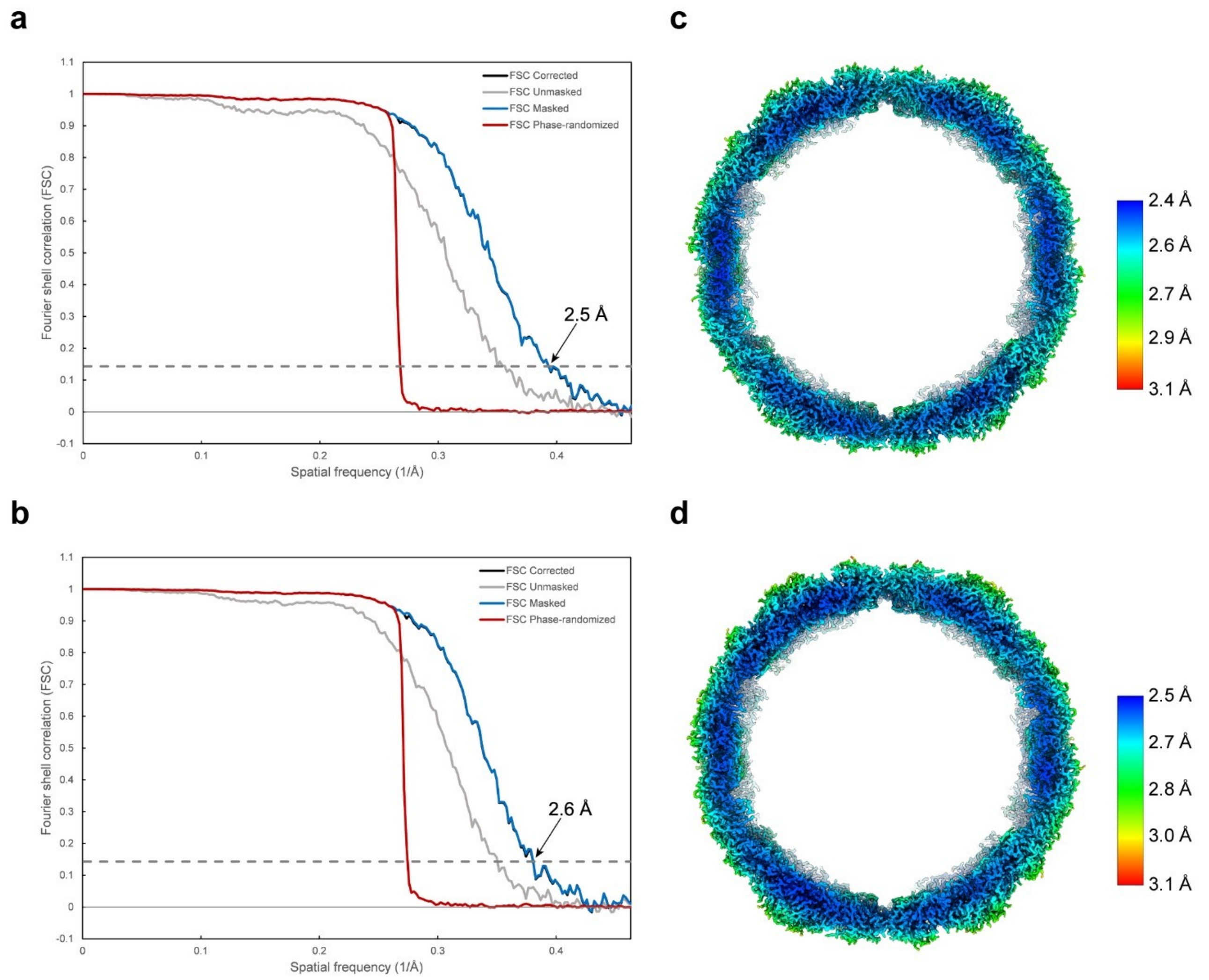

Supplementary Figure 1. Resolution analysis of the PV3-SC8$^{GPP3+GSH}$ and PV3-SC8$^{pleconaril+GSH}$ reconstructions. a,b Fourier shell correlation (FSC) calculated between two independent half sets of data as a function of spatial frequency is plotted for the PV3-SC8$^{GPP3+GSH}$ and PV3-SC8$^{pleconaril+GSH}$ reconstructions, respectively. FSC is plotted for the original unmasked half-maps (grey) and masked half-maps that had density corresponding to solvent removed (blue). FSC is also shown for phase-randomized half-maps (red) used to compensate for possible effects of the masking procedure before calculating the final corrected FSC (black). Good agreement between the masked and corrected curves indicated no adverse effects from the masking. The resolution at which the

corrected curve drops below the FSC=0.143 threshold (grey dashed line) is indicated with an arrow. **c,d** Local resolution analysis of the final cryo-EM electron potential maps for PV3-SC8$^{GPP3+GSH}$ and PV3-SC8$^{pleconaril+GSH}$ reconstructions, respectively as assessed by RELION local resolution estimation. A central slice through the VLPs is viewed along the icosahedral twofold axis and the distribution of local resolution (in Å) is shown coloured from blue to red according to the colour key shown.

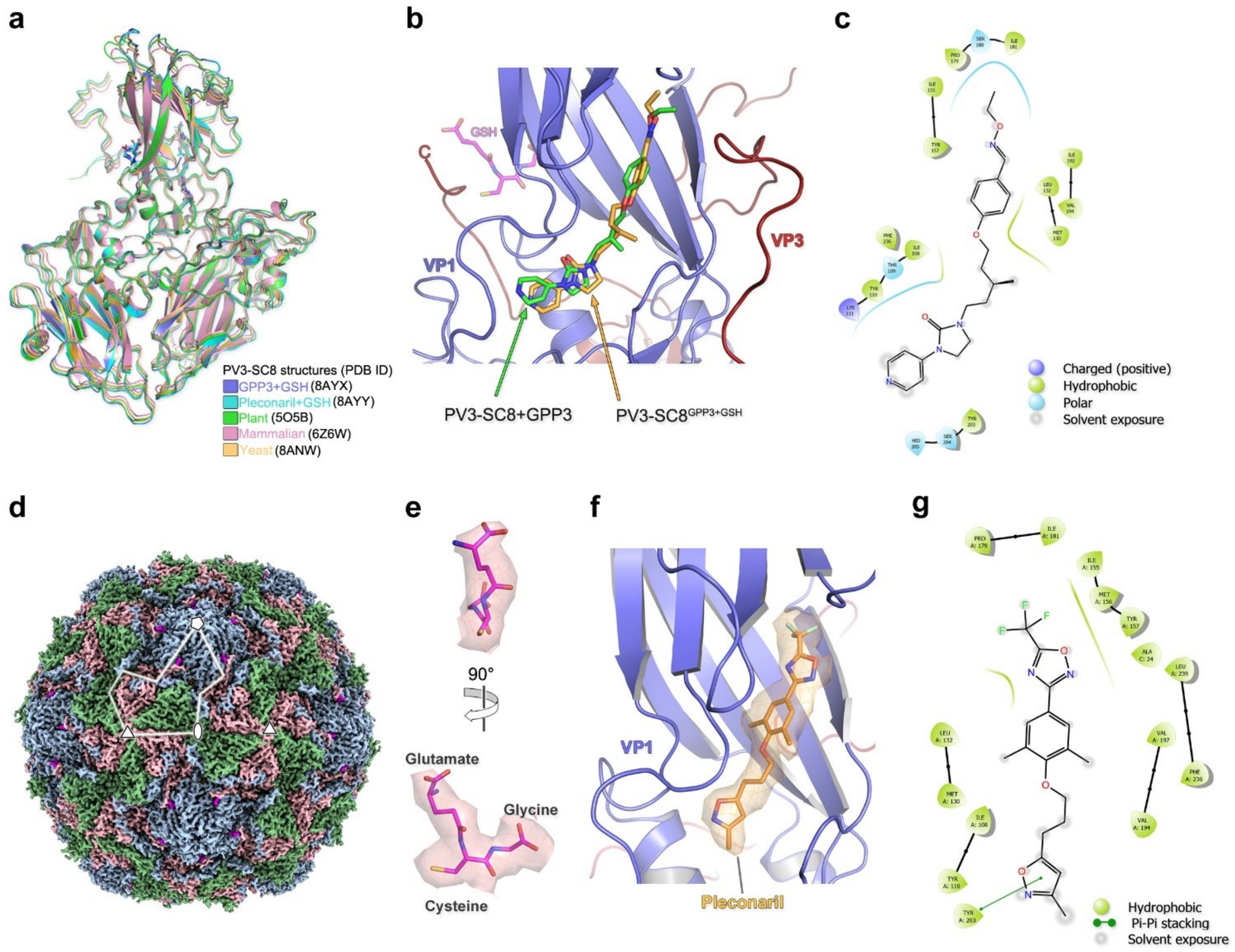

Supplementary Figure 2. Similarity of PV3-SC8 structures, PV3-SC8$^{pleconaril+GSH}$ reconstruction and pocket factor binding details. **a** Structural superposition of the capsid protomers of the yeast PV3-SC8$^{GPP3+GSH}$ complex (blue) with yeast PV3-SC8$^{pleconaril+GSH}$ (cyan), apo PV3-SC8 (yellow)[1], PV3-SC8 from plant cell expression (green)[2] and PV3-SC8 from mammalian cell expression (pink)[3]. **b** VP1 pocket of PV3-SC8$^{GPP3+GSH}$ comparing binding mode of bound GPP3 (orange sticks) with GPP3 bound in plant cell produced PV3-SC8[2] (green sticks, PDB ID 5O5P). **c** Ligand interaction diagram for GPP3 bound in the VP1 hydrophobic pocket of the PV3-SC8$^{GPP3+GSH}$ complex, generated using Schrödinger Maestro v12.9.137[4]. **d** Three-dimensional reconstruction of

PV3-SC8 VLP after incubation with a molar excess of GSH and pleconaril. The VLP is viewed along the icosahedral twofold symmetry axis with the VP1, VP0 and VP3 subunits of the capsid protomer coloured light blue, light green and light red, respectively, and GSH in magenta. A single kite-shaped capsid protomer is outlined in white and five, three and twofold symmetry axes labelled with symbols. **e** Cryo-EM electron potential map with GSH fitted as a stick model and elemental colouring for oxygen (red), nitrogen (blue) and sulphur (yellow). The map is displayed at a contour level of 1 σ. **f** Cartoon depiction of the VP1 hydrophobic pocket with bound pleconaril fitted as an orange stick model into the cryo-EM potential map (1.5 σ contour level). All maps are rendered at a radius of 2 Å around atoms. **g** Ligand interaction diagram for pleconaril bound in the VP1 hydrophobic pocket of the PV3-SC8$^{pleconaril+GSH}$ complex, generated using Schrödinger Maestro v12.9.137[4].

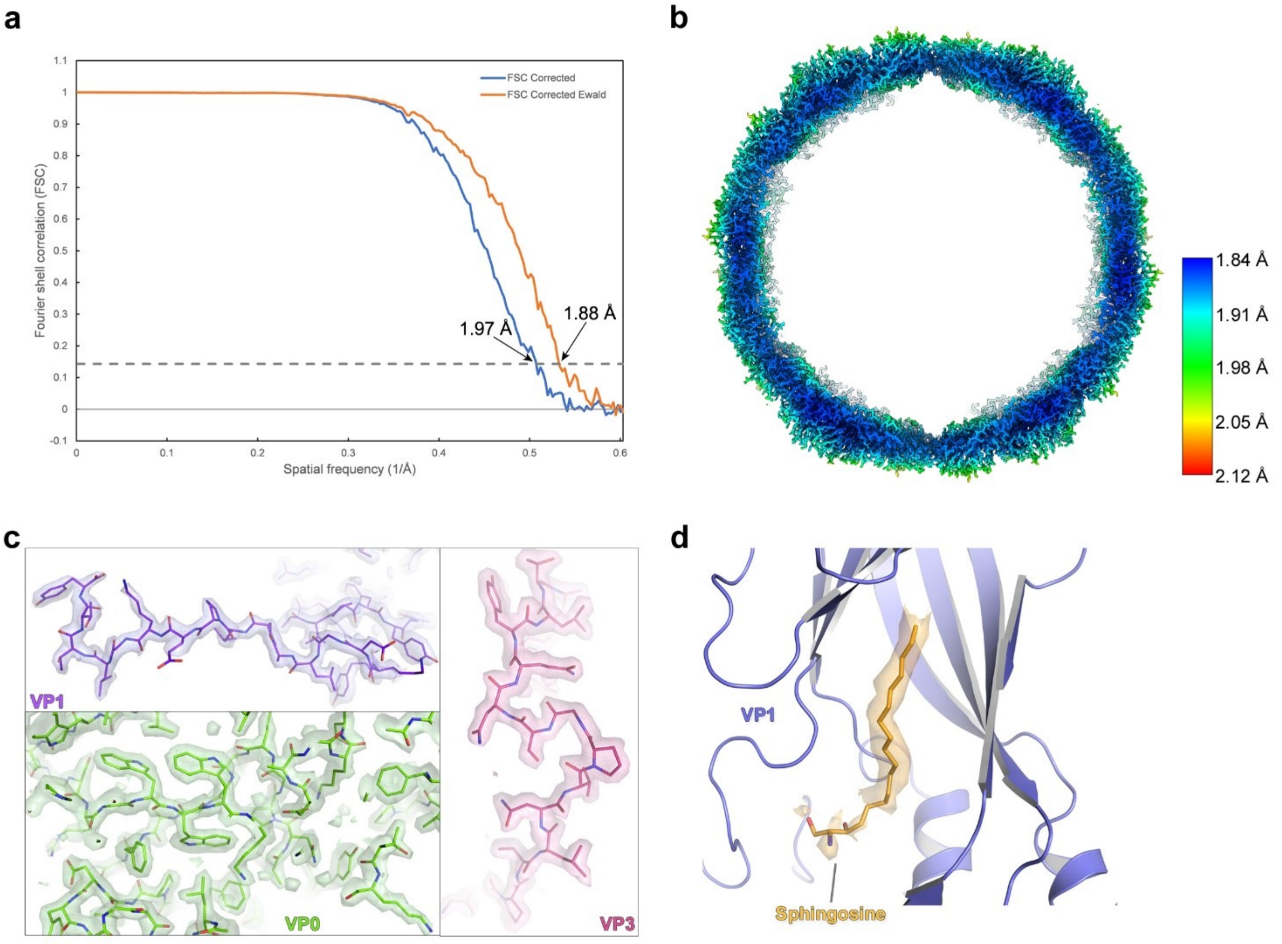

Supplementary Figure 3. Resolution analysis of the wt PV2-CP17 reconstruction before and after Ewald sphere correction and electron potential map features. a Fourier shell correlation (FSC) calculated between two independent half sets of data as a function of spatial frequency is plotted for the wt PV2-CP17 reconstruction. The final corrected FSC is plotted for reconstructions before (blue) and after (orange) Ewald sphere correction. The resolution at which the corrected curve drops below the FSC=0.143 threshold (grey dashed line) is indicated with an arrow. **b** Local resolution analysis of the final cryo-EM electron potential map for the wt PV2-CP17 reconstruction as assessed by RELION local resolution estimation. A central slice through the reconstruction is viewed along the icosahedral twofold axis and the distribution of local resolution (in Å) is shown coloured from blue to red according to the scale bar shown. **c** Representative snapshots of the cryo-

EM electron potential map for the VP1, VP0 and VP3 subunits of the wt PV2-CP17 reconstruction after Ewald sphere correction and sharpening with a *B*-factor of -15 Å^2. The map is displayed at a contour level of 2.5 σ. **d** Cartoon depiction of sphingosine modelled into the Cryo-EM potential map (contour level 1 σ) in the VP1 pocket of the wt PV2-CP17 reconstruction. All maps are rendered at a radius of 2 Å around atoms. The cryo-EM electron potential map around the hydrophilic head domain of the sphingosine was disordered.

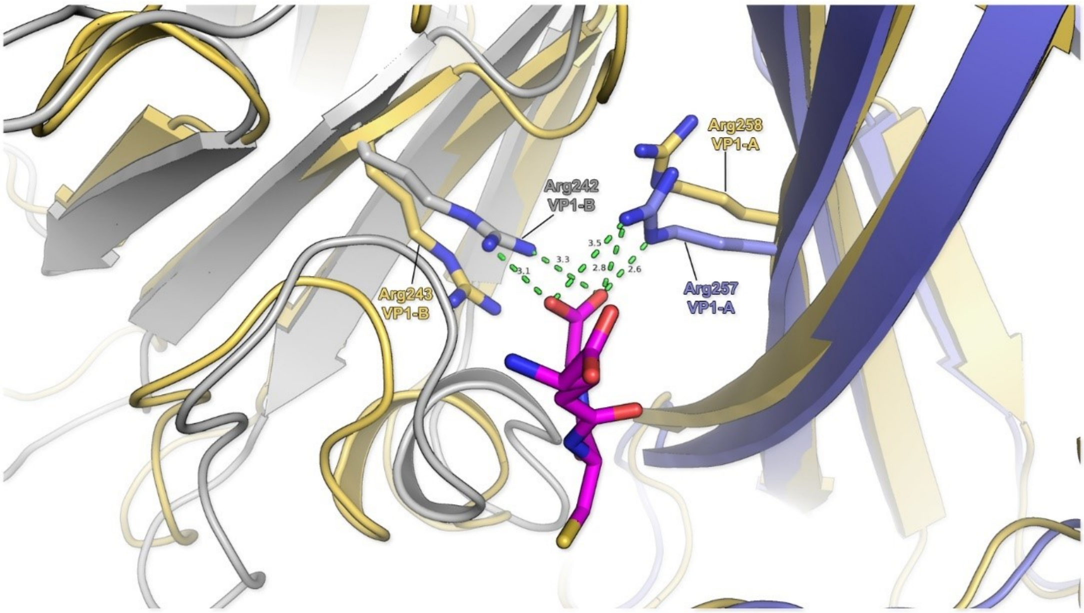

Supplementary Figure 4. GSH is not capable of binding expanded PVs in the C-antigenic conformation. Cartoon representation focussed on the GSH binding pocket of the PV3-SC8$^{GPP3+GSH}$ structure (PDB ID 8AYX) superposed onto the cryo-EM structure of the poliovirus type 1 (Mahoney strain) 135S-like expanded (C-antigenic) particle in complex with a monoclonal antibody[5] (PDB ID 6P9O). VP1 of protomer A and protomer B are coloured blue and grey, respectively, for PV3-SC8$^{GPP3+GSH}$, and pale gold for the PV1 C-antigenic particle. GSH is shown as a magenta stick model. The key arginine residues that stabilise the binding of GSH are shown as sticks, labelled, and the interactions distance with GSH shown as dashed green lines. Arginine-243 and arginine-258 in the expanded PV structure are displaced away from their respective positions in the GSH-bound structure, abrogating the ability of GSH to bind the interprotomer surface pocket in the C-antigenic conformation.

Supplementary References

1 Sherry, L. *et al.* Production and Characterisation of Stabilised PV-3 Virus-like Particles Using Pichia pastoris. *Viruses* **14**, 2159 (2022).

2 Marsian, J. *et al.* Plant-made polio type 3 stabilized VLPs-a candidate synthetic polio vaccine. *Nat Commun* **8**, 245, doi:10.1038/s41467-017-00090-w (2017).

3 Bahar, M. W. *et al.* Mammalian expression of virus-like particles as a proof of principle for next generation polio vaccines. *NPJ Vaccines* **6**, 5, doi:10.1038/s41541-020-00267-3 (2021).

4 Schrödinger Release 2021-3: Maestro Version 12.9.137, Schrödinger, LLC, New York, NY, 2021.

5 Shah, P. N. M. *et al.* Cryo-EM structures reveal two distinct conformational states in a picornavirus cell entry intermediate. *PLoS Pathog* **16**, e1008920, doi:10.1371/journal.ppat.1008920 (2020).
