# Supplementary material for: A conserved glutathione binding site in poliovirus is a target for antivirals and vaccine stabilisation
Source: Commun Biol. 2022 Nov 25;5:1293. doi: 10.1038/s42003-022-04252-5 (PMC9700776; doi:10.1038/s42003-022-04252-5)
Supplement: Supplementary file 3 — Description of Additional Supplementary Files [file 42003_2022_4252_MOESM3_ESM.pdf]

Description of Additional Supplementary Files

File name: Supplementary Data 1
Description: Source data underlying Fig. 4.
